# Postprandial triglyceride-rich lipoproteins-induced premature senescence of adipose-derived mesenchymal stem cells via the SIRT1/p53/Ac-p53/p21 axis through oxidative mechanism

**DOI:** 10.18632/aging.202298

**Published:** 2020-12-09

**Authors:** Qun-yan Xiang, Feng Tian, Xiao Du, Jin Xu, Li-yuan Zhu, Li-ling Guo, Tie Wen, You-shuo Liu, Ling Liu

**Affiliations:** 1 Department of Cardiovascular Medicine, The Second Xiangya Hospital, Central South University, Changsha 410011, Hunan, PR China; 2 Research Institute of Blood Lipid and Atherosclerosis, Central South University, Changsha 410011, Hunan, PR China; 3 Modern Cardiovascular Disease Clinical Technology Research Center of Hunan Province, Changsha 410011, Hunan, PR China; 4 Cardiovascular Disease Research Center of Hunan Province, Changsha 410011, Hunan, PR China; 5 Department of Geriatrics, Institute of Aging and Geriatrics, The Second Xiangya Hospital, Central South University, Changsha 410011, Hunan, PR China; 6 Department of Geriatric Cardiology, The First Affiliated Hospital of Zhengzhou University, Zhengzhou 450000, Henan, PR China; 7 Department of Emergency Medicine, The Second Xiangya Hospital, Central South University, Changsha 410011, Hunan, PR China; 8 Emergency Medicine and Difficult Disease Institute, The Second Xiangya Hospital, Central South University, Changsha 410011, Hunan, PR China

**Keywords:** triglyceride-rich lipoproteins, adipose-derived mesenchymal stem cells, premature senescence, SIRT1, oxidative stress

## Abstract

The accumulation of senescent adipose-derived mesenchymal stem cells (AMSCs) in subcutaneous white adipose tissue (WAT) is the main cause for the deterioration of WAT and the subsequent age-related disorders in obesity. The number of AMSCs staining positively for senescence-associated-β-galactosidase (SA-β-Gal) increased significantly after incubation with postprandial triglyceride-rich lipoproteins (TRL), accompanied by an impaired cell proliferation capacity and increased expression of inflammatory factors. Besides, the expression of anti-aging protein, silent mating-type information regulation 2 homolog 1 (SIRT1), was downregulated significantly, while those of acetylated p53 (Ac-p53), total p53, and p21 proteins were upregulated significantly during postprandial TRL-induced premature senescence of AMSCs. Furthermore, the production of intracellular reactive oxygen species (ROS) in the TRL group increased significantly, while pretreatment with the ROS scavenger N-acetyl-L-cysteine effectively attenuated the premature senescence of AMSCs by decreasing ROS production and upregulating SIRT1 level. Thus, postprandial TRL induced premature senescence of AMSCs through the SIRT1/p53/Ac-p53/p21 axis, partly through increased oxidative stress.

## INTRODUCTION

Adipose tissue is at the nexus linking the aging process and nutrient metabolism. Dramatic changes in fat mass, distribution, and function occur with advancing age, presenting as a reduction in the total mass of white adipose tissue (WAT), the redistribution of WAT from subcutaneous tissue to the abdominal cavity, and the deterioration of WAT function [1]. The main function of healthy subcutaneous WAT (sWAT) is to store excess energy as less active triglyceride (TG) in the form of lipid droplets. However, the amount of sWAT will decline during aging, accompanied by an impaired capacity for fat storage that leads to the increased release of free fatty acids (FFA) into circulation. These highly lipotoxic FFA overflow into non-subcutaneous sites, such as visceral fat, contributing to an increased susceptibility to visceral obesity, inflammation, and insulin resistance [2–4]. To some extent, aging of the body starts with sWAT senescence [5]. Therefore, the mechanism of sWAT senescence should be explored to prevent the occurrence and development of age-related disorders.

Adipose-derived mesenchymal stem cells (AMSCs), which account for 15–50% of the cells in WAT, can give rise to new adipocytes and sustain the normal function of WAT [6]. The accumulation of senescent AMSCs in sWAT is the main cause for the senescence of fat tissue and subsequent age-related disorders, manifesting as decreased proliferation and differentiation capacities, as well as increased lipotoxicity and inflammation in senescent AMSCs [7, 8]. Clearance of senescent AMSCs could prevent or delay age-related disorders and extend a healthy lifespan [9]. However, senescent AMSCs are not only found in elderly individuals, but also in young obese humans and mice [10, 11]. This kind of stress-induced senescence is termed as “premature senescence” to distinguish it from “replicative senescence”, which is mainly characterized by telomere shortening with advancing age [12]. Premature senescence in cells often occurs after exposure to various type of stress, such as FFA, hypoxia, hydrogen peroxide, or other physical and chemical stimuli [13]. It was reported that a high-calorie diet induced premature senescence of subcutaneous AMSCs in non-elderly patients [14]; however, the underlying mechanism is unclear.

Diet-induced obesity is usually accompanied by dyslipidemia, especially postprandial hypertriglyceridemia [15]. Elevated TG levels after a high-fat meal results in an increased number of circulating triglyceride-rich lipoproteins (TRL), including chylomicrons, very-low density lipoproteins, and their remnant particles [16]. Similar to insulin (INS), increased postprandial TRL are also considered as a key natural inducer of adipogenic differentiation [17], which is one of the important mechanisms of obesity. Although postprandial TRL induced premature senescence in endothelial progenitor cells [18], there has not been a study of their potential effect on premature senescence of subcutaneous AMSCs.

Silent mating-type information regulation 2 homolog 1 (SIRT1, sirtuin 1), a nicotinamide adenine dinucleotide (NAD^+^)-dependent deacetylase, has been implicated in a variety of physiological processes, including senescence, obesity, and inflammation [19, 20]. Significantly decreased SIRT1 level or activity was detected in premature senescent WAT of obese mice [21]. SIRT1 is an important deacetylase for the transcription factor p53. Decreased SIRT1 level or/and activity would result in reduced deacetylation of p53, and the subsequent upregulation of acetylated p53 (Ac-p53) [22]. Ac-p53 can bind to cyclin-dependent kinase and inhibit its activity, leading to increased p21 expression, cell cycle arrest, and ultimately, cellular senescence [23, 24]. SIRT1 is regulated by a number of factors, including oxidative stress [25]. In endothelial cells, TRL increased oxidative stress [26]; however, their influence on SIRT1 was not reported. In the present study, we aimed to investigate the role of postprandial TRL isolated from patients with hypertriglyceridemia in the senescence of mice subcutaneous AMSCs, and to further explore the potential mechanisms of the identified effects.

## RESULTS

### Postprandial TRL induced premature senescence in AMSCs during adipogenic differentiation

**Figure 1:**
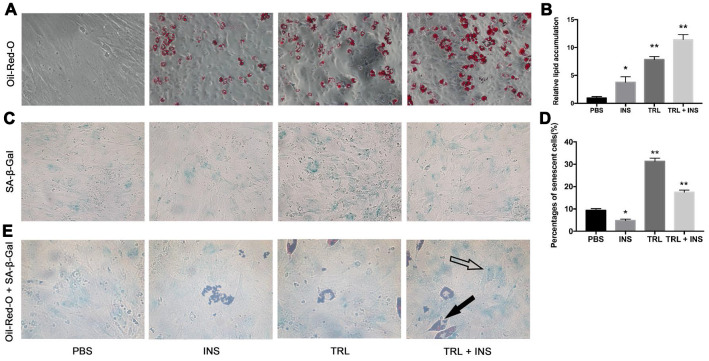
**Postprandial TRL induced both adipogenesis and premature senescence in AMSCs.** (**A**) AMSCs were treated with PBS, 10 μg/mL INS alone, 100 μg/mL TRL alone, or 100 μg/mL TRL + 10 μg/mL INS for 8 d after 48h confluence and then stained by Oil-Red-O. Images were obtained under a microscope (×200 magnification). (**B**) Quantification of relative lipid accumulation was measured for absorbance at 520 nm. (**C**) SA-β-Gal was performed to detect senescent cells. Images were obtained under a microscope (×200 magnification). (**D**) SA-β-Gal positive cells were counted manually by scanning a total of 200 cells in each sample. (**E**) SA-β-Gal positive cells were found in both undifferentiated (only blue SA-β-Gal in the cytoplasm, marked by a hollow arrow) and differentiating (both blue SA-β-Gal and red lipid droplets in the cytoplasm, marked by a solid arrow) AMSCs. Images were obtained under a microscope (×400 magnification). Data are expressed as mean ± SD (n ≥ 3). ^*^P < 0.05, ^**^P < 0.01 when compared with the PBS group.

Previously, we observed that postprandial TRL, co-incubated with INS, induced adipogenic differentiation of 3T3-L1 preadipocytes [17]. AMSCs from mice sWAT were incubated with 100 μg/mL postprandial TRL or 10 μg/mL INS, or both after 48 h confluence in the present study. At the end of 8 day (d), Oil-Red-O staining revealed that the AMSCs had been stimulated to differentiate into lipid-laden adipocytes with combination of postprandial TRL and INS, while fewer lipid droplets were found within cells incubated with TRL or INS alone, and almost no lipid droplets were observed in cells treated with phosphate buffered saline (PBS) (Figure 1A, 1B), indicating that TRL had a synergistic effect with INS on adipogenesis.

SA-β-Gal staining was used to detect senescent (i.e., SA-β-Gal positive) cells in four groups. The number of SA-β-Gal positive cells in the TRL group was higher than that in the TRL + INS group (Figure 1C, 1D), suggesting an inhibitory effect of INS on TRL-induced senescence in AMSCs. Intriguingly, Oil-Red-O and SA-β-Gal double positive cells were also detected (Figure 1E), indicating that senescence occurred not only in undifferentiated AMSCs, but also in differentiating ones. To eliminate both the disturbance of adipogenesis induced by TRL in combination with INS and the controversial role of INS in cellular senescence [27, 28], subsequent experiments were performed using AMSCs incubated with postprandial TRL alone to explore the role and potential mechanisms of senescence.

### Postprandial TRL induced premature senescence of AMSCs in a concentration-dependent manner

**Figure 2:**
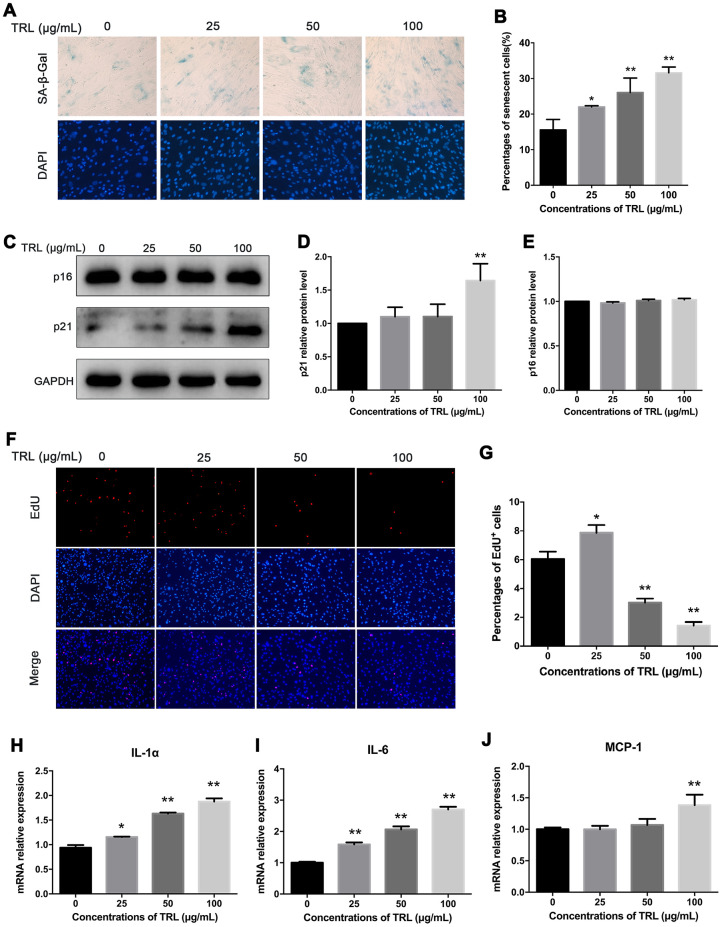
**Postprandial TRL induced premature senescence and SASP in AMSCs.** (**A**, **B**) AMSCs reached approximately 30%-40% culture-confluence were incubated with 0, 25, 50, or 100 μg/mL postprandial TRL for 8 d, and then SA-β-Gal (upper row) and DAPI (lower row) double staining was performed to detect the senescent cells and nuclei, respectively (**A**). Images were obtained under a microscope (×200 magnification). SA-β-Gal positive cells were counted manually by scanning a total of 200 cells in each sample (**B**). (**C**–**E**) Protein levels of senescent markers, p21 and p16, were detected using western blotting (**C**), and then the relative protein levels of p21 (**D**) and p16 (**E**) were analyzed using ImageJ. (**F**, **G**) The proliferation capacity of AMSCs incubated with different concentrations of postprandial TRL was measured using an EdU incorporation assay (**F**) and the EdU positive cells were counted using ImageJ (**G**). Images were obtained under a microscope (×100 magnification). (**H**–**J**) Expression levels of genes encoding senescence-related inflammatory cytokines, including IL-1α (**H**), IL-6 (**I**), and MCP-1 (**J**), were detected using qRT-PCR in AMSCs incubated with postprandial TRL at 0, 25, 50, or 100 μg/mL for 8 d. Data are expressed as mean ± SD (n ≥ 3). ^*^P < 0.05, ^**^P < 0.01 when compared with the control group.

AMSCs were stained by SA-β-Gal and 4’, 6-diamino-phenylindole (DAPI) after incubation with postprandial TRL at different concentrations (0, 25, 50, and 100 μg/mL) for 8 d. The number of senescent cells increased significantly as the concentration of postprandial TRL increased, indicating that postprandial TRL induced premature senescence in a concentration-dependent manner (Figure 2A, 2B). Besides, the level of the senescence marker p21 increased significantly in cells treated with 100 μg/mL postprandial TRL compared with that in the control group, whereas the level of p16 did not show any significant difference among the four groups (Figure 2C–2E). To explore the effect of postprandial TRL on the proliferation capacity of AMSCs, a 5-Ethynyl-2'-deoxyuridine (EdU) incorporation assay was performed. The proliferation capacity of AMSCs was inhibited significantly by 50 μg/mL and 100 μg/mL postprandial TRL, while it was improved by 25 μg/mL postprandial TRL when compared with the control group (Figure 2F, 2G). Moreover, 100 μg/mL postprandial TRL significantly increased the mRNA levels of genes encoding senescence-related inflammatory cytokines, including interleukin-1α (IL-1α), interleukin-6 (IL-6) and monocyte chemotactic protein (MCP-1) (Figure 2H–2J) These results suggested that high concentrations of postprandial TRL induced premature senescence and the senescence-associated secretory phenotype (SASP) in AMSCs.

To explore the adipogenic differentiation capacity of senescent AMSCs induced by postprandial TRL, AMSCs were incubated with cocktail inducers (standard adipogenic stimuli including insulin, IBMX and Dexamethasone) after pretreatment with PBS or postprandial TRL for 8 d, respectively. It was found that the cells with pretreatment of postprandial TRL produced significantly fewer lipid droplets than those treated with PBS. The results supported that postprandial TRL induced premature senescence of AMSCs, accompanied by an impaired adipogenic differentiation capacity (Supplementary Figure 1).

### Postprandial TRL induced premature senescence of AMSCs in a time-dependent manner

**Figure 3:**
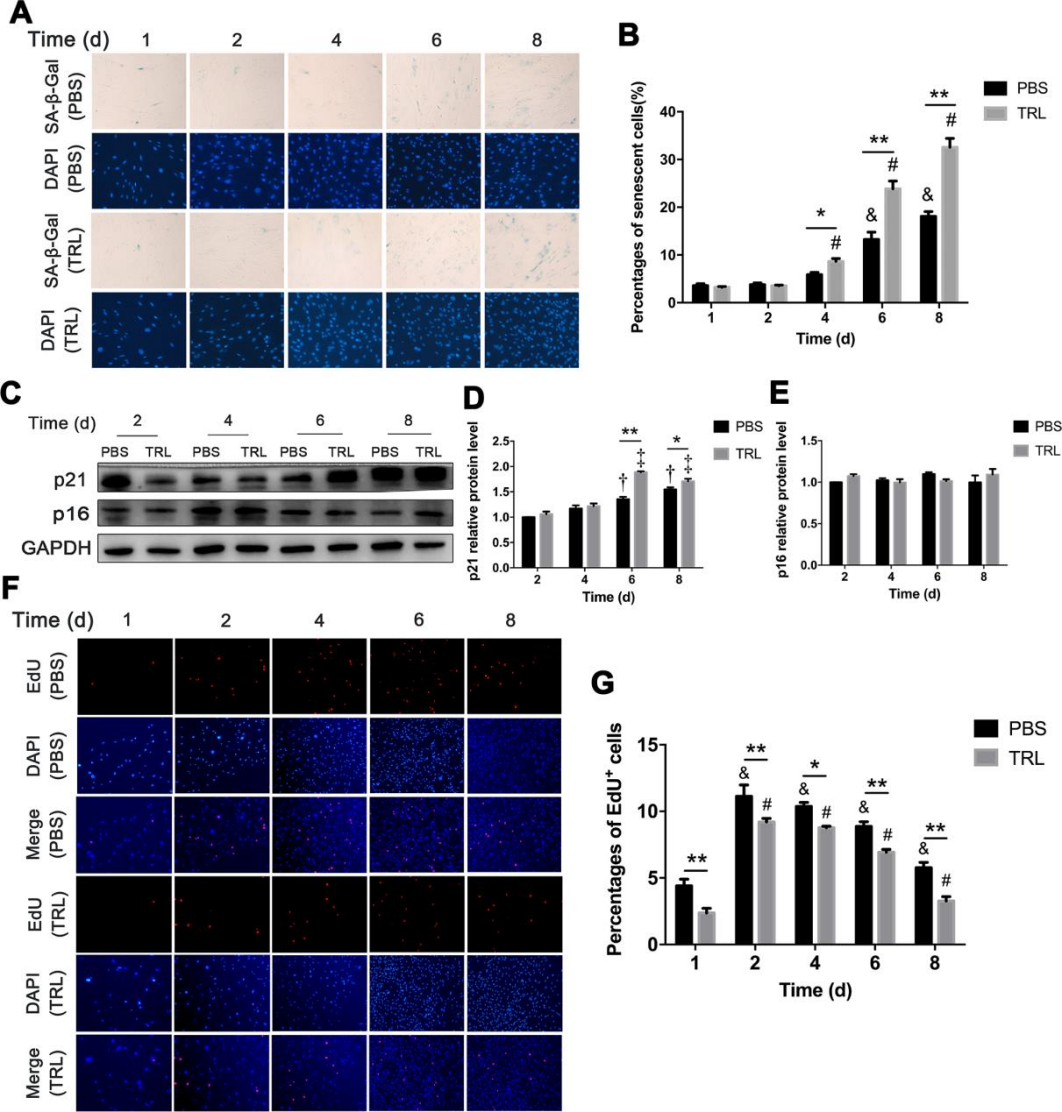
**Postprandial TRL induced premature senescence of AMSCs in a time-dependent manner.** (**A**, **B**) AMSCs reached approximately 30%-40% culture-confluence were incubated with PBS (two upper rows) or 100 μg/mL postprandial TRL (two lower rows) for 8 d, and then SA-β-Gal and DAPI staining was performed to detect the senescent cells and nuclei at day 1, 2, 4, 6, and 8 (**A**). Images were obtained under a microscope (×200 magnification). SA-β-Gal positive cells were counted manually by scanning a total of 200 cells in each sample (**B**). (**C**–**E**) Protein levels of p21 and p16 were detected using western blotting (**C**), and then the relative protein levels of p21 (**D**) and p16 (**E**) were analyzed using ImageJ. (**F**, **G**) The proliferation capacity of AMSCs incubated with PBS or 100 μg/mL postprandial TRL was measured using an EdU incorporation assay at day 1, 2, 4, 6, and 8, respectively (**F**) and EdU positive cells were counted using ImageJ (**G**). Images were obtained under a microscope (×100 magnification). Data are expressed as the mean ± SD (n ≥ 3). ^*^P < 0.05, ^**^P < 0.01 when compared with the PBS group on the same day, ^&^*P* < 0.05 when compared with the PBS group at day 1, ^#^*P* < 0.05 when compared with the TRL group at day 1, ^†^*P* < 0.05 when compared with the PBS group at day 2, ^‡^*P* < 0.05 when compared with the TRL group at day 2.

SA-β-Gal staining showed that the number of senescent AMSCs increased significantly from day 4 to day 8 in the TRL group, and from day 6 to day 8 in the PBS group. Moreover, there were significantly more senescent AMSCs in the TRL group than in the PBS group at day 4, 6, and 8 (Figure 3A, 3B). Similarly, significant upregulation of p21 level, but not p16 level, was observed, and the difference in p21 level between the TRL group and the PBS group reached statistical significance from day 6 to day 8 (Figure 3C–3E). Although the proliferation capacity of AMSCs in both the PBS group and the TRL group was enhanced significantly from day 2 when compared with their baseline capacities, respectively, postprandial TRL (100 μg/mL) significantly inhibited the proliferation capacity of AMSCs compared with that of PBS (Figure 3F). Collectively, these results indicated that postprandial TRL induced premature senescence of AMSCs in a time-dependent manner.

### Changes in the SIRT1/p53/Ac-p53/p21 axis in postprandial TRL-induced premature senescence of AMSCs

**Figure 4:**
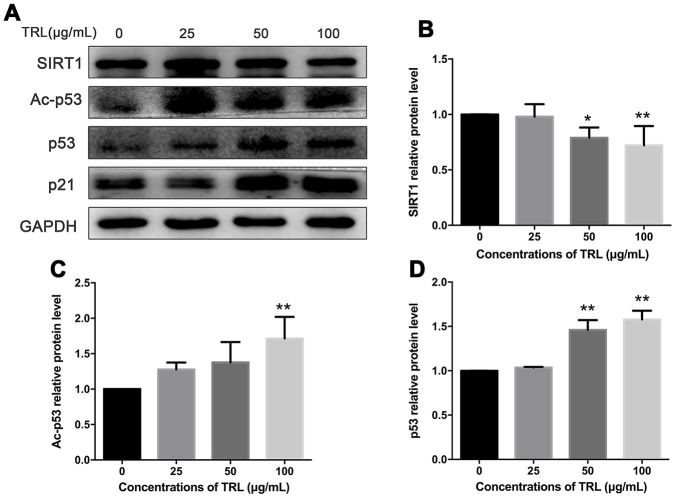
**Changes in the SIRT1/p53/Ac-p53/p21 pathway in postprandial TRL-induced AMSC senescence.** (**A**) AMSCs reached approximately 30%-40% culture-confluence were treated with 0, 25, 50, or 100 μg/mL postprandial TRL for 8 d, and were harvested to detect the protein levels of SIRT1, Ac-p53, p53, and p21 using western blotting. (**B**–**D**) The relative protein levels of SIRT1 (**B**), Ac-p53 (**C**), and p53 (**D**) were analyzed using ImageJ. Data are expressed as the mean ± SD (n ≥ 3). ^*^P < 0.05, ^**^P < 0.01 when compared with the control group.

The SIRT1/p53/Ac-p53/p21 axis is a typical senescence regulatory pathway. As a well-known anti-senescence protein, SIRT1 was downregulated significantly in AMSCs treated with postprandial TRL at 50 or 100 μg/mL, accompanied by significant upregulation of p53, Ac-p53, and p21 protein levels. However, 25 μg/mL postprandial TRL did not change the level of these proteins significantly (Figures 4A–4D, 2C, 2D). These data suggested that the SIRT1/p53/Ac-p53/p21 pathway was involved in regulating postprandial TRL-induced senescence of AMSCs.

### N-acetyl-L-cysteine (NAC) inhibited postprandial TRL-induced AMSCs premature senescence and reactive oxygen species (ROS) production

**Figure 5:**
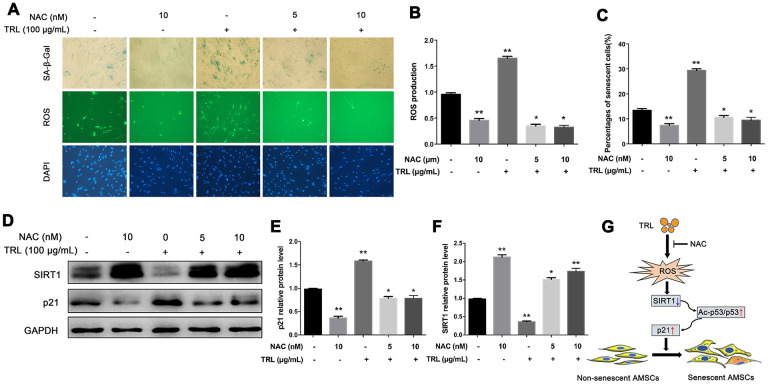
**Antioxidant NAC alleviated postprandial TRL-induced AMSC senescence and ROS production.** (**A**) AMSCs reached approximately 30%-40% culture-confluence were treated with PBS, NAC (10 nM), TRL (100 μg/mL), or TRL (100 μg/mL) with pretreatment of 5 or 10 nM NAC for 8 d. Subsequently, the intracellular ROS production and the number of senescent cells were evaluated using the fluorescent probe, DCFA-DA (green under fluorescence microscope), and SA-β-Gal staining (blue under the light microscope), respectively. Nuclei were stained using DAPI (blue under the fluorescence microscope). Images were obtained under a microscope (×200 magnification). (**B**) The fluorescence intensity analysis of ROS production. (**C**) SA-β-Gal positive cells were counted manually by scanning a total of 200 cells in each sample. (**D**–**F**) the protein levels of p21 and SIRT1 were detected using western blotting (**D**), and then the relative protein levels of p21 (**E**) and SIRT1 (**F**) were analyzed using ImageJ. Data are expressed as the mean ± SD (n ≥ 3). ^*^P < 0.05, ^**^P < 0.01 when compared with the PBS group. (**G**) A schematic illustration of the proposed mechanism of AMSC premature senescence induced by postprandial TRL. Postprandial TRL increased intracellular oxidative stress, downregulated SIRT1 level, and activated the p53/Ac-p53/p21 pathway, which ultimately promoted the premature senescence of undifferentiated and differentiating AMSCs.

Increased oxidative stress is involved in the process of cell senescence [18, 29, 30]. In this study, we found that intracellular ROS production in the TRL group was higher than that in the PBS group (Figure 5A, 5B). This indicated that an oxidative mechanism could be associated with postprandial TRL-induced premature senescence of AMSCs. Pretreatment of AMSCs with 5 or 10 nM NAC, an antioxidant, markedly decreased ROS production (Figure 5A, 5B), accompanied by a decreased number of SA-β-Gal positive AMSCs (Figure 5C) and downregulated level of p21 (Figure 5D, 5E). Meanwhile, the level of SIRT1 was restored by NAC pretreatment (Figure 5D, 5F). Taken together, these results suggested that increased oxidative stress might promote postprandial TRL-induced AMSCs senescence.

## DISCUSSION

In the present study, we found that postprandial TRL induced premature senescence of subcutaneous AMSCs, accompanied by impaired cell proliferation and differentiation capacity, and increased levels of inflammatory factors. Mechanistically, the SIRT1/p53/Ac-p53/p21 pathway was partly involved in regulating this process. Moreover, we demonstrated that intracellular ROS production increased during postprandial TRL-induced premature senescence of AMSCs, and antioxidants such as NAC was an efficient approach to prevent premature senescence of AMSCs by upregulating SIRT1 protein level.

Senescent AMSCs have an impaired differentiation potential, in addition to a decreased proliferation capacity [31]. Recently, we observed that the adipogenesis induced by postprandial TRL and INS was at a relatively low-grade compared with that induced by cocktail inducers *in vitro* [17, 32, 33]. On the one hand, this indicated that adipogenesis could be induced by those natural inducers to a lesser extent *in vivo*. On the other hand, it was reasonable to hypothesize that there was a relationship between adipogenesis and senescence during the treatment of preadipocytes with postprandial TRL. In this study, the senescent cells were derived from both undifferentiated and differentiating AMSCs, accompanied by increased levels of senescence markers and inflammatory factors. Therefore, postprandial TRL might not only induce adipogenesis but also promote the senescence of AMSCs *in vivo*.

In the present study, we noted a rapid effect of high concentrations of postprandial TRL on AMSCs senescence. Roldan et al. [10] reported that AMSCs isolated from non-elderly obese individuals showed a senescent phenotype when they were cultured *in vitro* without additional intervention to at least passage 7, which was suggestive of premature senescence. However, senescent cells were also found in AMSCs at passage 4 when treated with postprandial TRL at 100 μg/mL for 4-8 d, although the AMSCs were obtained from mice with normal weight. Undoubtedly, AMSCs underwent premature senescence after incubation of postprandial TRL. Interestingly, postprandial TRL at low concentrations induced fewer senescent cells with increased proliferation capacity of AMSCs. With increasing concentrations of postprandial TRL, the number of senescent cells gradually increased, accompanied by an impaired proliferation capacity. To some extent, postprandial TRL at high concentrations caused a sustained and chronic depletion of AMSCs in WAT. Conversely, postprandial TRL at lower concentrations could be helpful to maintain the stemness of AMSCs.

Anti-aging protein SIRT1 is closely associated with the occurrence of senescence, including premature senescence. Emerging evidence indicated that SIRT1 level was downregulated significantly in WAT of obese mice induced by a high-fat diet, which was related to excess intake of nutrients and subsequent decreased NAD^+^ biosynthesis [34, 35]. Similarly, in the present study, the protein level of SIRT1 decreased significantly in AMSCs incubated with postprandial TRL at high concentrations. It was reported that postprandial TRL could be endocytosed via the LDL receptor family and could upregulate the level of key enzymes related to lipolysis within preadipocytes [17], which suggested that the content of intracellular nutrients, such as TG and FFA, might derive from internalized TRL. This indicated that the excessive intake of nutrients could promote the premature senescence of AMSCs in WAT, manifesting as downregulation of SIRT1 level and upregulation of p21 level [10, 36].

Indeed, different concentrations of postprandial TRL seemed to exert different impacts on SIRTI in this study. Postprandial TRL at the lowest concentration (i.e., 25 μg/mL) did not downregulate SIRT1 level. This suggested that postprandial TRL at low concentrations might play a relatively weak role in promoting the premature senescence of AMSCs. Increased protein level or acetylation of p53 is closely associated with the senescent phenotype. Excessive calorie intake led to the accumulation of oxidative stress in the adipose tissue of mice or patients with type 2 diabetes, and promoted senescence-like changes, such as increased SA-β-Gal activity and p53 level; meanwhile, inhibiting p53 activity in adipose tissue markedly ameliorated the senescence-like changes [11]. The increasing amounts of TRL particles, especially in the postprandial state, is a sign of excess energy intake. Postprandial TRL at high concentrations markedly downregulated SIRT1 level, meanwhile upregulating p53 and p21 levels, which was slightly different from the effect of high glucose, which accelerated premature senescence in fibroblasts, but did not change p53 level [37]. This indicated that high concentrations of postprandial TRL promoted the premature senescence of AMSCs through affecting the levels of SIRT1 and p53.

The protein level and activity of SIRT1 can be regulated by the redox status [13]. Oxidants derived from cigarette markedly decreased SIRT1 level in lung epithelial cells and accelerated cellular senescence [38]. Moreover, the potent oxidant, hydrogen peroxide, induced the senescence of human lung fibroblasts by impairing SIRT1 activity and accumulating Ac-p53 [22]. It was found that postprandial TRL, which had been hydrolyzed by lipoprotein lipase, produced a variety of oxidative products in endothelial cells, of which the most abundant one was 13-hydroxy octadecadienoic acid [26]. Increased 13-hydroxy octadecadienoic acid was able to inhibit protein kinase C and trigger the phosphorylation and nuclear exportation of forkhead box O3, leading to the downregulation of antioxidant enzymes, and finally, cellular senescence [39, 40]. Thus, postprandial TRL-induced oxidative stress might also participate in decreasing SIRT1 expression or activity, or both.

As a type of membrane penetrating antioxidant, NAC was reported to ameliorate the premature senescence of other cells by restoring SIRT1 protein level or activity, and decreasing Ac-p53 protein level [41–43]. In the present study, the protein level of SIRT1 was significantly restored with NAC pretreatment. More importantly, NAC not only inhibited the ROS production, but also attenuated 100 μg/mL postprandial TRL-induced AMSCs premature senescence, which supported the view that postprandial TRL at high concentrations might induce AMSC senescence at least partly through an oxidative mechanism.

The present study had several limitations. Firstly, the activity of SIRT1 was not detected. Secondly, the content of FFA and lipid peroxide were not measured. Thirdly, considering the relationship between ROS production and mitochondrial function, the potential effect of postprandial TRL on mitochondrial function was not addressed, which will be explored in our future study.

In conclusion, we found that postprandial TRL induced premature senescence of AMSCs, supporting the hypothesis that postprandial TRL was a key inducer for senescence of adipose tissue in diet-induced obesity.

Mechanistically, as shown in the schematic illustration in Figure 5G, postprandial TRL increased intracellular oxidative stress, and then downregulated SIRT1 level and activated the downstream p53/Ac-p53/p21 pathway, which ultimately promoted the premature senescence of AMSCs. These findings not only provide an explanation for high-fat diet-induced premature senescence in AMSCs, but also indicated a direction for therapies to prevent and treat certain obesity-related disorders.

## MATERIALS AND METHODS

### Cell culture

Primary mice AMSCs were isolated from inguinal subcutaneous adipose tissue as described previously with minor modifications [44]. Briefly, freshly excised inguinal fat pads from male C57/BL6 mice, 7–10 d old, were minced and digested with 0.1% collagenase type I (Gibco, Grand Island, NY, USA) for 45 min at 37° C. After neutralization, the floating adipocytes were discarded by centrifugation at 1000 g for 5 min. Then, the remaining pellet was resuspended and filtered through 100 μm nylon filter mesh and plated in Dulbecco’s Modified Eagle’s Medium/Nutrient Mixture F-12 (DMEM/F-12) with low glucose supplemented with 10% fetal bovine serum (FBS), 100 U/ml penicillin, 100 U/ml streptomycin (complete medium, all from Gibco) and incubated at 37° C in a 5% CO_2_ humidified atmosphere. The culture medium was refreshed every 2 d and cells were passaged with trypsin/ethylenediaminetetraacetic every 2–3 d. Cells at passage 4 were used in subsequent experiments.

### Postprandial TRL preparation

Postprandial TRL were isolated using our previously described method [45]. In short, blood samples were collected from patients with hypertriglyceridemia at 4 h after a high-fat meal, and then postprandial TRL was separated using density gradient ultracentrifugation (d < 1.006 g/mL), dialyzed against PBS, and concentrated. The concentration of postprandial TRL was determined using a bicinchoninic acid (BCA) protein assay kit (CWBIO, Beijing, China) and subsequently sterilized through a 0.22 μm microporous filter.

### Adipogenic differentiation and Oil-Red-O staining

After approximately 48 h confluence, AMSCs at passage 4 were used for the adipogenesis assay (designated day 0). After 8 d, the cells were fixed with 4% paraformaldehyde for 30 min and stained with 0.3% Oil-Red-O (Sigma, St. Louis, MO, USA) at room temperature for 30 min [17]. After lipid droplets were visualized under a light microscope, the Oil Red O in cells were extracted by 100% isopropyl alcohol and measured for absorbance at 520 nm.

### SA-β-Gal staining

The senescent cells were verified using a SA-β-Gal staining kit (Beyotime, Jiangsu, China), as described previously [46]. Briefly, AMSCs were washed twice with PBS and fixed in β-galactosidase fixation solution (2% formaldehyde/0.2% glutaraldehyde in PBS) for 15 min. Then, the cells were washed three times with PBS and incubated in SA-β-Gal staining solution (pH 6.0) overnight at 37° C. The numbers of SA-β-Gal positive AMSCs were counted manually from a total of 200 cells in each sample under a light microscope.

### DAPI staining

After SA-β-Gal staining, the cells were washed once with PBS and counterstained with DAPI (Sigma) for 5 min to count the total cell number under a fluorescence microscope.

### Cell proliferation assay

The cellular proliferation capacity was measured by EdU staining using an EdU Cell Proliferation Kit with Alexa Fluor 594 (Beyotime). Briefly, the cells were incubated with EdU for 2 h at 37° C/5% CO_2_. After incubation, the cells were washed with PBS to remove the free EdU probe and then fixed in 4% paraformaldehyde at room temperature for 30 min before being stained with DAPI for 5 min. After an additional wash in PBS, the cells were observed under a fluorescence microscope. The numbers of EdU positive and DAPI positive cells were counted automatically using ImageJ software version 1.52k (NIH, Bethesda, MD, USA).

### ROS production

Intracellular ROS production was measured using a ROS kit (Beyotime) according to the manufacturer’s instructions. Briefly, after incubation with PBS, NAC (10 nM), TRL (100 μg/mL), or TRL (100 μg/mL) with pretreatment of 5 or 10 nM NAC for 8 d, the AMSCs were washed twice with PBS and incubated with 10 μM 2,7-dichlorodihydrofluorescein diacetate (DCFH-DA) for 20 min at 37° C. Then, the cells were washed three times with serum-free DMEM/F-12 and photographed under a fluorescence microscope at an excitation wavelength of 488 nm and an emission wavelength of 525 nm. The fluorescence intensity was analyzed using ImageJ.

### Western blotting analysis

Protein levels were detected using Western blotting analysis, as previously described [47]. Briefly, cells were lysed in Mammalian Protein Extraction Reagent (Thermo Fisher Scientific, Waltham, MA, USA) and the protein concentration was assayed using a BCA kit (CWBIO, Beijing, China). Equal amounts (10 to 20 μg) of protein from each sample was subjected to SDS-PAGE and transferred onto a poly-vinylidene difluoride membrane. The membranes were blocked for 2 h in PBS containing 5% skim milk and 0.1% Tween-20, and then incubated overnight at 4° C with the following primary antibodies: anti-SIRT1 (9475S, Cell Signaling Technology, Danvers, MA, USA, 1:1000), anti-Ac-p53 (2570S, Cell Signaling Technology, 1:2000), anti-p53 (2524T, Cell Signaling Technology, 1:1000), anti-p21 (10355-1-AP, Proteintech, Rosemont, IL, USA, 1:1000), anti-p16 (ab211542, Abcam, Cambridge, MA, USA, 1:1000), and anti-GAPDH (GB11002, Servicebio, Wuhan, China, 1:1000). The membranes were then washed with PBS containing 0.1% Tween-20 and incubated with horseradish peroxidase-conjugated goat anti-rabbit (ZB-2301, ZSGB-BIO, Beijing, China, 1:5000) or goat anti-mouse (SA-00001-1, Proteintech, 1:5000,) secondary antibodies at room temperature for 1 h. The immunoreactive protein bands were visualized using an enhanced chemiluminescence substrate (Advansta, Menlo Park, CA, USA) and quantified using ImageJ. The relative target protein level was normalized to that of glyceraldehyde-3-phosphate dehydrogenase (GAPDH).

### RNA isolation and quantitative reverse transcriptase polymerase chain reaction (qRT-PCR)

Total RNA from AMSCs was extracted using GeneJET RNA Purification Kit (Thermo Fisher Scientific) as described previously [48]. The first strand cDNA was synthesized from equal amounts of total RNA in each sample using a cDNA Synthesis Kit (Thermo Fisher Scientific). Quantitative real-time PCR (qPCR) was then performed using the cDNA as the template with the SYBR Green Master Mix (Thermo Fisher Scientific) in an ABI 7300 Real-Time PCR System (Applied Biosystems, Foster City, CA, USA), according to manufacturer’s instructions. The relative expression of mRNA was calculated using the comparative CT (2^-ΔΔCt^) method, and normalized to that of GAPDH. The primers used in this study are shown in Supplementary Table 1.

### Statistical analysis

Data were represented as the mean ± standard deviation (SD). And were analyzed using GraphPad Prism software version 7.0. Student’s t-test was used to compare two different groups, while one-way analysis of variance (ANOVA) was used for multiple groups. A level of *P* < 0.05 was considered statistically significant. All experiments were repeated at least three times, and representative experimental results are shown in the figures.

## Supplementary Material

Supplementary Figure 1

Supplementary Table 1
