# Supplementary material for: Postprandial triglyceride-rich lipoproteins-induced premature senescence of adipose-derived mesenchymal stem cells via the SIRT1/p53/Ac-p53/p21 axis through oxidative mechanism
Source: Aging (Albany NY). 2020 Dec 9;12(24):26080–94. doi: 10.18632/aging.202298 (PMC7803527; doi:10.18632/aging.202298)
Supplement: Supplementary Figure 1 [file aging-12-202298-s001.pdf]

SUPPLEMENTARY FIGURE

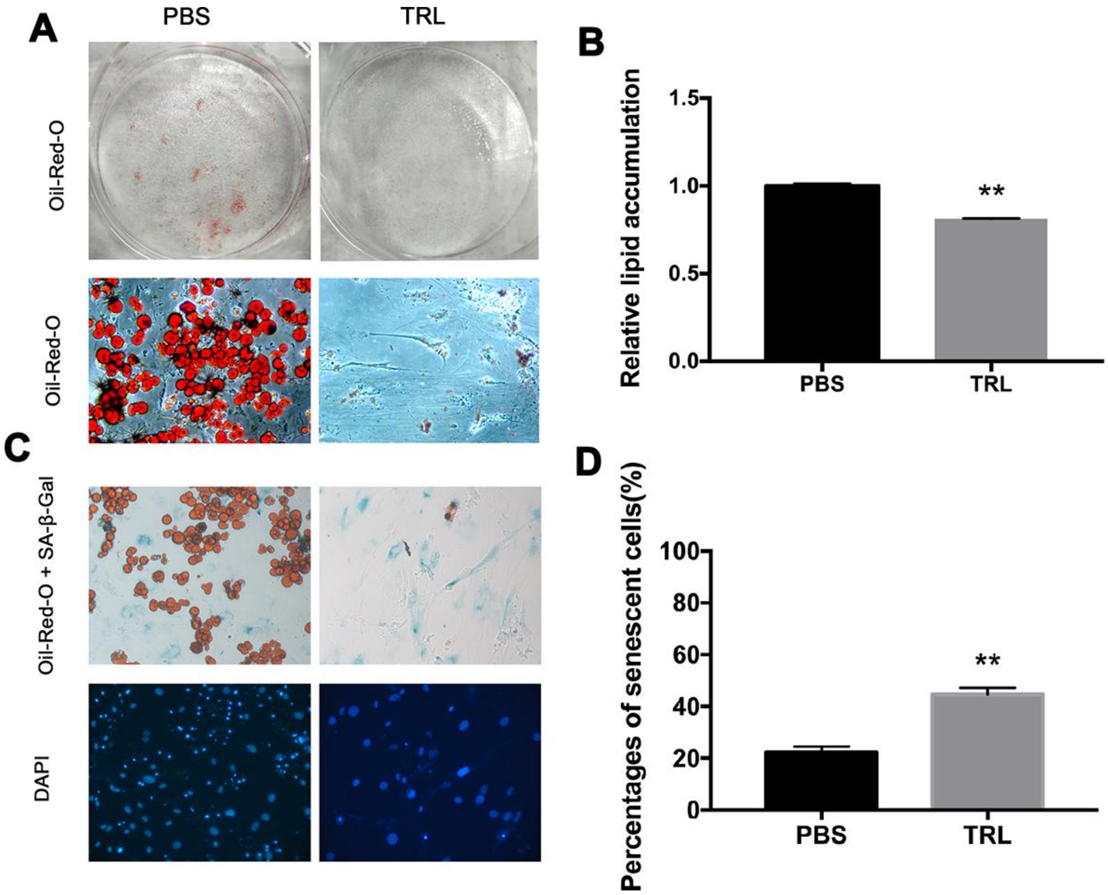

Supplementary Figure 1. The adipogenic differentiation capacity induced by an adipogenesis-stimulating cocktail decreased after AMSCs were pretreated with postprandial TRL. (**A**) Lipid droplets detected by Oil-Red-O staining in cocktail-induced AMSCs with pretreatment of PBS or postprandial TRL (100 μg/mL) for 8 d. (**B**) Quantification of relative lipid accumulation was measured for absorbance at 520 nm. (**C**) AMSCs were stained using both SA-β-Gal and Oil-Red-O staining. (**D**) SA-β-Gal positive cells were counted manually by scanning a total of 200 cells in each sample. Images were obtained under a microscope (×200 magnification). $^{**}P < 0.01$ when compared with the PBS group.
