# Supplementary material for: Postprandial triglyceride-rich lipoproteins-induced premature senescence of adipose-derived mesenchymal stem cells via the SIRT1/p53/Ac-p53/p21 axis through oxidative mechanism
Source: Aging (Albany NY). 2020 Dec 9;12(24):26080–94. doi: 10.18632/aging.202298 (PMC7803527; doi:10.18632/aging.202298)
Supplement: Supplementary Table 1 [file aging-12-202298-s002.pdf]

SUPPLEMENTARY TABLE

Supplementary Table 1. The primer sequences used in this study.

Gene	Primer sequence (5'-3')
GAPDH	Forward: AAGGTCATCCCAGAGCTGAA
	Reverse: AGGAGACAACCTGGTCCTCA
IL-1α	Forward: CGGGTGACAGTATCAGCAACGT
	Reverse: ATGACAAACTTCTGCCTGACGAG
IL-6	Forward: TGTATGAACAACGATGATGCA
	Reverse: AGGACTCTGGCTTTGTCTTTCT
MCP-1	Forward: TCACCTGCTGCTACTCATTCACCA
	Reverse: TACAGCTTCTTTGGGACACCTGCT
